# Manufacturing a *Monster*: an autoethnographic analysis of enforced isolation, objectification and the destruction of self

**DOI:** 10.3389/fpsyt.2025.1694605

**Published:** 2025-10-27

**Authors:** Alexis Quinn

**Affiliations:** Restraint Reduction Network, London, United Kingdom

**Keywords:** enforced isolation, autoethnography, objectification, autism, trauma, therapeutic relationships, human rights, psychiatric care

## Abstract

**Introduction:**

This perspective article examines the impact of enforced isolation on the authors sense of Self. The research explores how systemic objectification and the blocking of vital “mirroring” within seclusion and long-term segregation (LTS) in psychiatric hospitals in England can lead to the erosion of Self. The paper posits that enforced isolation is not therapeutic, but a destructive intervention rooted in neuronormative ideology that ultimately escalates distress, prolonging detention.

**Methods:**

The autoethnographic perspective offers a qualitative understanding of experience to examine the phenomena of isolation and trauma. The reflexive analysis is rooted in the author’s lived experiences of repeated and enduring exposure to seclusion and LTS.

**Results:**

Enforced isolation eroded the author’s Self due to systemic objectification and a lack of positive “mirroring”. Consequently, the Self could only be sustained through perverse connections with staff e.g., shared negative emotions such as fear, aggression and hate. With a Self-reconfigured around negative affect, the lines are blurred between intimacy and aggression, resulting in shattering implications for the author’s ability to have relationships and love.

**Discussion:**

Enforced isolation is positioned as a destructive intervention, manufacturing rather than containing, distress. This perspective reframes isolation from a clinical tool to a harmful practice, contradicting therapeutic goals. Aligning with wider research, the paper calls for a transformative shift towards rights-based, relational models, such as HOPE(S), that prioritize human connection to prevent iatrogenic harm.

## 1 Introduction - the crisis of enforced isolation in psychiatric settings

Enforced isolation of autistic individuals and/or people with learning disabilities in psychiatric settings is not merely a clinical practice but can be understood as a systemic human rights crisis demanding urgent re-evaluation (1–6). Enforced isolation is often framed as a necessary risk-management tool (7, 8), but research consistently finds it is a destructive intervention that fundamentally dismantles the human psyche by attacking the relational nature of its existence (5, 6, 9, 10). Decades of high-quality research confirm human responses to isolation result in psychological trauma, creating the very behaviors it seeks to contain e.g., anxiety, loss of control, heightened sensory sensitivity, psychological regression, self-mutilation, rage, paranoia, cognitive dysfunction and withdrawal (5, 9–11). A systemic review by Chieze et al. (12) identified deleterious effects with post-traumatic stress was reported in up to 47% of individuals, highlighting isolations risk of causing high iatrogenic harm.

Internationally, human rights frameworks such as the *United Nations Standard Minimum Rules for the Treatment of Prisoners (the Nelson Mandela Rules)* (13) and the *UN Convention on the Rights of Persons with Disabilities (UNCRPD)* (1) challenge the legitimacy of enforced isolation. They reason it is a *prima facie* breach of international law and instead advocate for approaches that promote autonomy, mutual recognition and proactive, community-based care. In England, this international consensus is mirrored in multiple, recent high-level reports confirming that the issues are not anecdotal but symptoms of deep-seated systemic failure. The Care Quality Commission’s (CQC) 2020 report, ‘Out of sight – who cares?’, identified hospital environments exacerbate autistic distress and that seclusion and long-term segregation^1^ (LTS) are a direct consequence of failing to provide person-centered care (2). The House of Commons Health and Social Care Committee Report similarly described a system “horrendously failing autistic people”, reinforced by the CQC’s 2022 follow-up report (3). Subsequently, in 2023, the government-commissioned an Independent Review into 191 cases, unequivocally finding enforced isolation “has no therapeutic benefit” (4). In 2025, NHS England’s independent report into 122 cases of LTS was published, finding the practice has “devastating” consequences for autistic people and those with learning disabilities (6). These reports provide an irrefutable indictment of the status quo in hospitals in England, illustrating that the continued use of enforced isolation represents a catastrophic failure in person-centered care rather than isolated instances of individual malpractice.

This paper argues that enforced isolation manufactures distress, increasing risk and prolonging detention. I trace this destructive process as being rooted in neuronormative ideology: applying normative judgment to psychological states and viewing certain ways of being (e.g., non-autistic) as normal and ideal, while framing others (such as being autistic) as inherently defective (14). While related to ableism, involving broader discrimination against disability, neuronormativity is a specific ideology that privileges one neurotype over others and provides the justification for oppressive practice. It is this neuronormative ideology that gives rise to what I term *cultural restraint* - a pervasive, often implicit, system of beliefs and organizational structures that subtly objectify and dehumanize individuals, denying legal capacity and physical and mental integrity. Enforced isolation can be understood as a manifestation of cultural restraint e.g., a type of systemic dehumanization where authentic reactions to overwhelming environments are pathologized and met with force greater force/isolation. This calls into questions dominant clinical narratives, such as those presented in Tromans et al. (7, 8), which suggest patients sabotage staff attempts to end isolation and enjoy being in sensory and socially deprived spaces because they prefer to be alone/in control. In reframing such understandings/experiences through the lens of iatrogenic harm we can begin the challenge the predictable consequences of systemic failure.

While government reports and academic studies can catalogue the systemic nature of this crisis, they often cannot penetrate the walls of isolation to capture its vivid internal damage; they can count the days spent locked in a room but cannot articulate the experience of the Self^2^ unravelling within it. Therefore, to understand the mechanisms by which isolation manufactured a ‘*monster*,’ a different methodology is required, allowing for exploration of systemic issues through the raw immediacy of lived experience. Autoethnography (AE) facilitates reveals the phenomenological reality that statistics alone cannot convey and has therefore been adopted.

My journey into the psychiatric system was abrupt. Within a week, I went from being a schoolteacher to an object of clinical scrutiny, referred to by my room number - “Patient-11” (15). My autistic ways of coping with the death of my brother were reframed through a neuronormative lens as pathological, trapping me for four years in twelve different inpatient units. In a cycle of escalating *distress-coercion* (16–18), my involuntary reactions to the chaotic, sensory-assaulting environments were consistently objectified as *challenging behavior* (19) and met with force. This intensified my distress, leading to greater uses of force to contain me, further escalating distress and coercive measures, and so on. Ultimately, this cycle was only broken when I escaped detention to live in Africa (20). Situating my personal narrative within the broader political and clinical context, abstract policy failures and their devastating human consequences might be examined from within their lived context.

## 2 An autoethnographic perspective

Jack Henry Abbot, a man forcibly isolated in America said, “Solitary confinement can alter the ontological makeup of a stone” (9, p. xi). The institutional preoccupation with my biology precipitated a regression to an infantile state, where my needs were reduced to medication, feeding and toileting. Locked in a room, profound social deprivation created an ontological deficit - a crisis of being - where my sense of Self dissolved. Without being perceived it becomes difficult to perceive myself, and I felt compelled to verify my own existence: I threw my body against concrete walls to attain feedback. Based on this experience my methodological premise rejects any notion of a singular, objective reality, positing instead that experience is a “relational idiom of power” (21, p. 13) and that my identity/understandings are therefore forged within relational dynamics and permissions granted by others (22).

Grounded in a critical, relational constructionist framework, this AE perspective lays bare a concern that this work risks being dismissed as I may be construed an unreliable due to my diagnostic and patient status (23). That said, in understanding this vulnerability to epistemic invalidation we highlight the asymmetrical power imbalance upon which domination depends (24). With a commitment to reflexive visibility, I aim to present my “wounds, scars and hard-won understandings” (25, p. 331) so that truth may be fostered between my Self and the reader, generating a counter-narrative to power-laden accounts that frequently silence patient voice.

This AE fuses data with method by way of an iterative, reflexive process and the material for reflection is drawn from multiple sources e.g., embodied memories, journals, personal artwork and my own clinical notes (from the period). Rather than approaching these as objective facts, my sources served as catalysts for heuristic immersion (26) - a deep dwelling within the experiences they evoked, allowing for an integration of memory, reflection and theory. As such, academic literature was not applied to the narrative *a priori* but woven into my immersive processes to aid conceptual framings/understandings.

As both researcher and the researched, ethical considerations involved a continuous process of self-assessment regarding the risk of re-traumatization, supported by ongoing personal therapy, reflexive journaling and clinical supervision. Reflexivity was operationalized through these same mediums, creating a critical dialogue to examine bias and challenge narrative distortions, going after that which is ungraspable (26).

In the following AE sections, I draw directly on my embodied memories which triggered my psychic and physiological destruction. First, I describe my immediate response to the barren, escape-proof environment, mirroring decades of research showing the abrupt collapse of cognitive and relational capacities, consistent with *isolation panic* (27). Next, I convey how the denial of recognition ignites a destructive arc of shame and rage, which in turn gave rise to a perverse form of connection - a state of loving-hate, manufacturing a *‘Monster’* - my ‘afterlife’ continues to be defined by these relational patterns. These sections demonstrate the alignment between my lived experience and the wider literature that underlines the systemic, predictable harms of enforced isolation and its traumatic legacy.

## 3 The destructive arc

*(Author’s note: Sections 3, 4, and 5 contain descriptions of psychological distress and are included to explore the phenomenology of isolation, central to the analysis).*

As staff moved me towards the seclusion room, I was flooded with unbearable affect, my skin feeling as though it was on fire. Extreme arousal, coupled with staff bodies pressing down on me, meant any connection to thought, imagination or external reality, vanished. My “I” literally dropped out in *autistic meltdown* (28), and what took over were seemingly instinctual drives insisting on immediate muscular action; I screamed, flailed, kicked and hit, my body apparently doing its best to survive without a guiding self-consciousness.

Exhausted, in my concrete box - slightly larger than a parking space, I sat on a blue mattress, on the rubber floor and looked through what felt like a postage-stamp sized window. Observing staff members in the hallway holding a clipboard, I was aware of my being the object of staff’s writing activity, while to myself, I had interiority. High up, in the corner of the windowless box, additional 24/7 vision-based monitoring intrusively scrutinized my every action.

Psychologically naked, with my perceived defects exposed, my instinctual attempts to self-soothe through stimming and other sensory-modulatory activity e.g., pacing, spinning and rolling, were viewed through the lens of neuronormativity and recited weekly to a multi-disciplinary team. This created an impossible paradox: the very behaviours that provided me with the ability to self-regulate were the ones that increased my distress and staff concern, justifying my continued confinement – I had to stop.

Cultural restraint manifested a new source of shame relating to my being overcome by traitorous temptations and demonstrating a *truly heteronymous will* (29) - that is betraying my Self to express the will of staff. Additionally, my basic needs, such as feeding and self-care, were posited as prototypical merging experiences to which staff were the answer e.g., staff were the breast, and my Self as hunger; staff were containing, and I, passively held. This was associated with a *shame of existence* (30). Cultural restrictive messaging blurred the lines between the inside and outside of my body as I internalized neuronormative, medical narratives “I am a dangerous – a *Monster* that needs this room”. Were these thoughts my own? This boundary violation was made concrete through invasive physical and chemical attacks and intensified by shattered privacy and sensory deprivation.

24hr lighting bounced from pale, windowless walls, intensifying boundarilessness; I only knew the time based on the food I was fed on the same floor I was forced to excrete upon. Ashamed, I engaged in furious self-criticism, which curdled into resentment and retaliatory rage … followed by furious self-criticism and then rage, indicative of a *shame-rage cycle* (31). In meltdown, staff would enter the room ramming me into the floor and injecting me with powerful antipsychotics. Consumed by guilt, I would withdraw into subservience, attempting to maintain or achieve neuronormative expectations. My inability to do so deepened my unbearable shame, which I came to anticipate. For instance, the mere sound of footsteps or jangling keys would cause my heart to race and palms to sweat, flooding me with fear of impending humiliation … and so I raged, and then felt unbearable shame…. Again.

### 3.1 The perverse dance for recognition

The profound trauma associated with isolation was not simply my inability to act, but that when I did act, no relief ensued. When staff position themselves as the ultimate power, refusing the fundamental human need for mutual recognition, people are forced into a domination-submission relationship (32). I became trapped in a perverse dance where my only means of recognition was through aggression (in restraint) or through submission (in adhering to a neuronormative behavioral agenda).

Paradoxically, in both domination and submission my deep aloneness and *social pain* (33) were alleviated. Social pain shares some of the same neural pathways with physical pain and when staff hands touched my own in gentle ways, or whether I had 10 men restraining me, I felt the agony ease with the whisper of alluring redemption and/or the fantasy of intimacy. Psychologically, I achieved mastery over my stimuli through *sensual gratification* (34) from the cruel mixture of anxiety and anticipation (24). For instance, in compliance, I felt enacted into a fictional care and treatment situation, cast to demonstrate my voluntary cooperation in *getting well* – here, staff witnessed progress, rewarding me with crayons, paper and food; in restraint care was framed in coercion, increasing its emotional intensity, allowing me to feel something in the nothingness.

### 3.2 The destruction of self

Each time I was confined for both short frequent periods e.g., in seclusion, or longer periods in LTS, staff could not mirror positive aspects of my Self e.g., acceptance, respect and love, leading to a catastrophic failure in my Self-structure. The more I was confined the greater and more irreversible the blocking of vital, life-giving mirroring necessary for a full, cohesive Self (35), speaking to isolations dose dependent harms (5, 9, 10).

My unmet needs for care and recognition resulted in more of my positive affective existence becoming *quasi-dead* and *dissociated* from my awareness (36). Any other loss e.g., that of my arm or leg, would have resulted in outcry, yet the murdering of large parts of my Self struck as silently as if it was of no consequence. As humans do not pre-exist their interactions but emerge through and as part of their entangled intra-relating (37), isolation acts as the ultimate denial of this reality. It is an attempt to create a separate object where an intra-connected Self once was. Fearing the complete termination of my Self, I felt a profound vacuum anxiety. If I was not perceived by an Other, how could I perceive my Self? Throwing myself against the walls to check I was still alive, my existence was being entirely restructured around the image staff procedurally and sensorially reflected back at me – the wanton, bad *Monster*.

## 4 Loving-hate – the creation of a ‘*Monster’*

My *Monster* was not just an internal defense, but an identity manufactured by the system to justify its actions. This manufacturing process was driven by the causal chain of cultural restraint e.g., neuronormativity, clinical documentation (reports, graphs and charts), staff behavior (reframing my instinctual survival reactions during meltdown as “spontaneous aggression” and “sensory driven acts of dangerousness”) and policies (restraint, behavioral modification and enforced isolation), directly producing the psychological consequences of objectification and identity fragmentation.

Staff were perhaps responding with *their own threat-based emotions* (38) creating a form of *malignant alienation* (39, 40) - a process where I was actively cast as a negative object whom staff postulated would be better off not existing. By constructing me as a *Monster*, staff could rationalize their use of overwhelming force as they were unable – partly due to the walls of isolation – to respond with compassion. In isolation my flesh became my total reality, and my affective life had grown so meagre. Consequently, opportunities to be known by an Other in more nuanced and positive ways progressively diminished because there was simply less of my Self available.

Unmercifully, the core of the Self cannot be destroyed (41). Because humans are neurobiologically wired to connect (42) and both staff and I were unable to provide recognition to one another, we escalated our efforts to find and possess the Other (32). In the face of ongoing empathic failure, my Self was forced to reorganized around a perverse relational dynamic - shared fear, anger and hate with staff - *loving-hate* (43). This adaptive relational structure meant intimacy, and therefore Self-survival, could only be achieved through the development of a hate-filled persona – my *Monster*.

Loving-hate meant negative affect (e.g., fear, anger and hate) dominated all relating, and its shared demonstration with staff provided powerful confirmation of our existence for one another. As such, I unconsciously sought out these perverse interactions to feel alive and sustain my Self. However, moments of perverse merging/connection (e.g., during restraint) were inevitably followed by withdrawal from staff which felt like catastrophic annihilation. Adding to this dynamic was that the very hand that fed and *cared* for me was also the hand that restrained me. This paradoxical attachment explains how I simultaneously detested staff for the harm they inflicted, while desperately needing them for the very recognition that guaranteed my Self-survival (both psychological and physiological). Perverse relating prolongs *enslavement* (32) and my experience is in keeping with research that demonstrates isolation is harder to end, the longer it progresses (5, 6).

## 5 My “Afterlife” – managing the *Monster*

Enforced isolation has left me feeling as though I have a darkness – a hole in my soul - that cannot be filled. Each day I experience the incessant re-enactment of its perverse dynamics where dependency activates shame, impotence and a lack of control, leading to symbolic re-staging of myself as a *stranger* (44) in relationships. This occurs because people show me aspects of my Self e.g., acceptance, trust and love, which I no longer recognize as being a part of me. I desperately seek to cast light into the darkness, trying to recognize both myself and Others as *not all bad* (e.g., in Klein’s depressive position) (45) and accept the complexities of shared humanity.

Yet, almost 10 years on, being with people in day-to-day unscripted situations feels impossible, not just difficult. Navigating and allowing others to represent my subjectivity in relationships involves a profound fear of recognition - I do not want Others to see me and what I certainly lack. Therefore, any encounter risks triggering long-established object ties and narrative truths: “am I really a *Monster*? If I am unable to love, am I evil?”. Conversely, I question, “If people are showing me kindness/love and I am not the *Monster* staff said I am, then who am I”? Additionally, I remain unclear whether a hand extended in name of care is actually caring or could harm me.

Feeling destabilized, I attack. Objects remain possessed and controlled in loving-hate and I berate myself for it, reinforcing internalized Self objectification. At the same time, I bewilderingly feel thirsty for more companionship and fear asking for it, anticipating people coming to learn of my monstrosity and withdrawing, or worse, I harm Others.

## 6 Discussion - towards rights-based, human-centered care

The preceding AE narrative illustrates how enforced isolation can become a destructive process that fundamentally undermines humanity, contradicting goals of *ethical health care* (46). My experiences, in keeping with current government reporting and the *UNCRPD* (1), suggest the need to view isolation as a violation of personal integrity and degrading treatment. I suggest that when psychiatric professionals confine a person, they implicitly tamper with relational meanings pertaining to power and recognition.

Connection is fundamental to the human condition (42), and so it is unsurprising that isolation escalates existing trauma and creates trauma its own, forcing a person into pathological adaptations by seeking recognition through perverse means. I struggled to leave isolation, not because I was sabotaging reintegration into ward settings (7), but because my relational system had been predictably rewired by isolation (32, 47, 48). This process is the logical outcome of a system restrained by a neuronormative straitjacket - a form of cultural restraint - that manufactures and enlivens through its ideology and distress-coercion/shame-rage cycles, the very *Monsters* it seeks to contain. For me, the antidote has been genuine recognition, where people have welcomed dependency, expanding my sense of Self and identity. Urgent statutory reform capable of influencing systemic change on the ground is required where models of care might be experienced as relational and rights based.

The *HOPE(S)* model, created by Dr Jennifer Kilcoyne and Danny Angus in high secure settings in England, offers compelling evidence that relational care is effective in getting people out of LTS (6). As cited in an external evaluation of the program commissioned by NHS England into 122 cases of isolated people, staff became more “solution focused” and “unstuck” from fear-driven practices when engaging with people in mutually meaningful ways. For instance, when patients experienced increased access to fresh air (up by 83.84%) and activities they found meaningful (up by 152.64%) there was a reduction in restrictive practices (e.g., physical restraint decreased by 21.19%, chemical restraint by 12.35% and seclusion by 33.43%); in 29% cases, individuals were discharged directly from isolation into the community.

Crucially, HOPE(S) was found to build “trust and hope through meaningful connections” with practitioners “role-modelling close, creative, fun and respectful interactions” that helped individuals feel “humanized” and “safe enough to be themselves again” (6). This is further supported by positive impacts on staff, who reported significantly improved compassion satisfaction and reduced burnout and secondary traumatic stress, enabling a shift from fear-based to compassionate care. When staff cultivate an inner sense of safety in themselves and the people they support, research shows a reduction in reliance on coercion as a means of managing staff’s own feelings of threat, demonstrating the potential of compassionate care (49).

The potential for evidence-based compassionate care (e.g., HOPE(S)) invites a painful but necessary reflection on how my journey might have been different had systems embraced human intra-connection rather than enacting a culturally restrictive *illusion of separateness* (37). A relational model of care might have meant staff met my distress with compassionate presence, understanding that the Self emerges through compassionate reciprocity, not isolation. Such an intra-connected response could have nurtured my terrified Self, subverting the development of the perverse ‘loving-hate’ dynamic born from a desperate need for recognition. My ‘afterlife’ might then have been a journey towards (re)weaving my thread which had come lose after the death of brother into the fabric of the whole, rather than a constant struggle to tame a now loser thread owing to a system-manufactured *Monster*.

The time for passive acceptance of the status quo is over. We must heed Eden’s (50) poignant warning, “if people are not fed love on silver spoons, they will learn to lick it from knives”. This is a call to action for a transformative shift in psychiatric practices, demanding that we prioritize human rights, relational ethics and an intimate understanding of the Self in our pursuit of compassionate services. Drawing from key actions outlined in recent independent reviews (2–4), I suggest reform must include:

- Mandated relational and rights-based training e.g., all staff trained in therapeutic, human rights-based care, including de-escalation and the HOPE(S) model.
- Abolition of enforced isolation, independent oversight and accountability e.g., enforced isolation must be recognized as a ‘never event’ for children and young people as well as those with disabilities such as autism and learning disabilities, triggering a serious investigation in the event of its use and holding management accountable. All instances must be subject to independent national oversight.
- Strengthening safeguarding and access to advocacy e.g., people in isolation must have immediate access to independent specialist advocacy and legal advice, and families must be invited to visit and raise concerns without obstruction.
- Meaningfully including people with lived experience in the design, delivery and oversight of services/policies to ensure they are fit for purpose.
- Investing in community-based alternatives e.g., moving away from episodic, crisis-driven detention to funding robust, community-based support, including ‘intensive recovery pods’, to prevent admissions.

Remember, the cold, objectless environment I was forced to occupy did not contain a *Monster*, it manufactured one. This phrase is not mere rhetoric! It is symbolic of the system’s capacity to deform psychic structure and identity through the coercive denial of something as fundamental as human connection.

## 7 Limitations

This autoethnographic perspective insights based on my subjective experience to achieve resonance, not generalizability. I have attempted to make transparent my personal biases, shaped by my trauma and the ways this inevitably influences my narrative focus and interpretation of events. The goal is not to present an objective or detached account but rather to share my understandings of the internal damage caused by enforced isolation and for these truths to exist between the reader and me. My account relies on embodied memories of traumatic events; although these are vivid, memory is a reconstructive process, undoubtedly influenced by my subsequent reflections and understandings. These limitations bind the scope of this paper, positioning it as one truth among many. It is intended to illuminate the human reality behind statistical and clinical data on the harms of enforced isolation and invite further research to complement and test these findings on a broader scale.

## Figures and Tables

**Figure 1:**
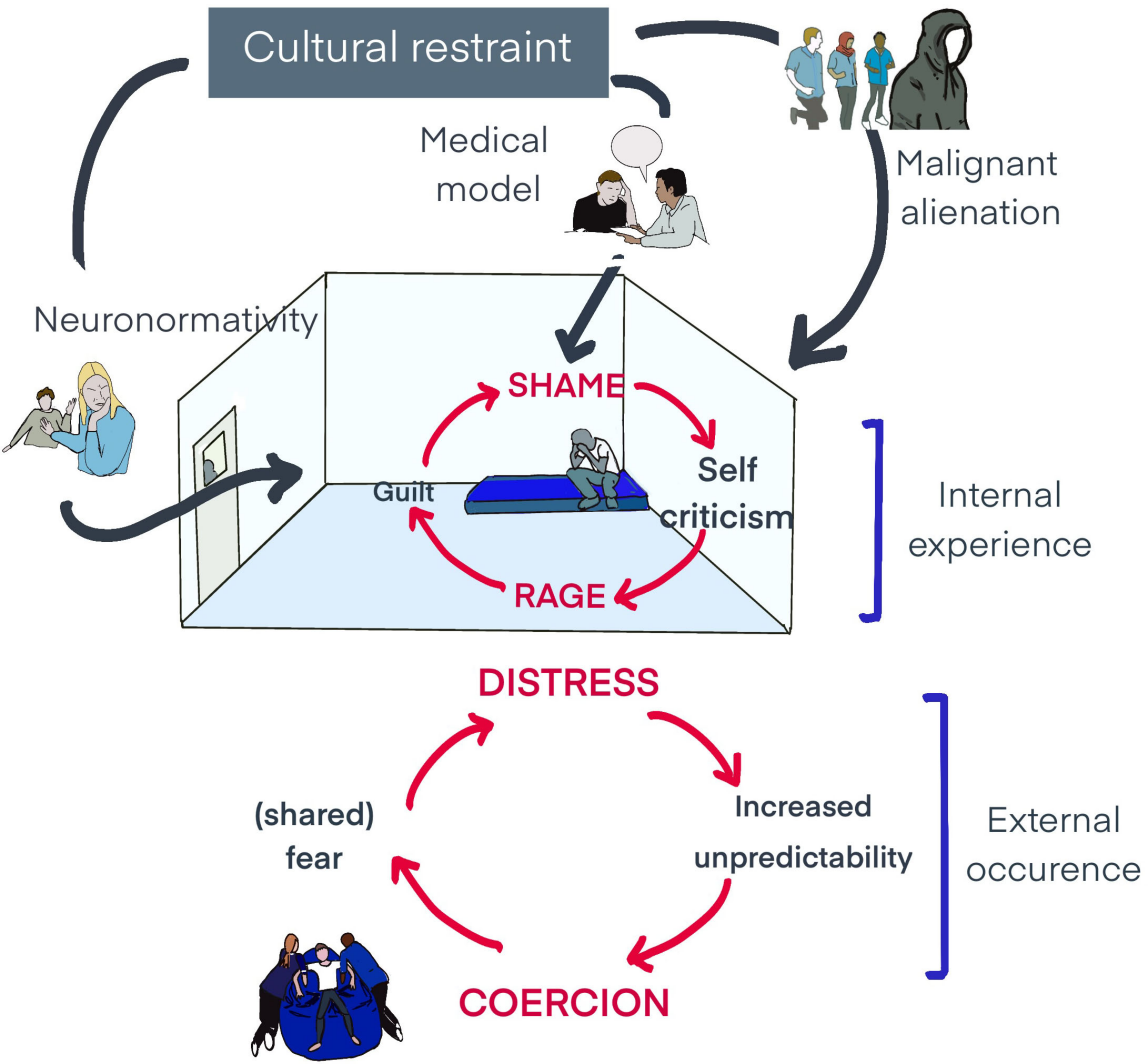
Interplay of cultural restraint, shame-rage and the distress-coercion cycle: This composite figure illustrates the cultural factors that instigate shame and distress resulting in greater occurrences of rage and therefore coercive practices. The figure visually demonstrates how concepts of neuronormativity and over medicalization (associated with cultural restraint) objectify a person, leading to internal processes of shame and rage, while externally, through a focus on negative forms of relating e.g., fear and observable distress, coercive measures perpetuate a destructive cycle.

**Figure 2:**
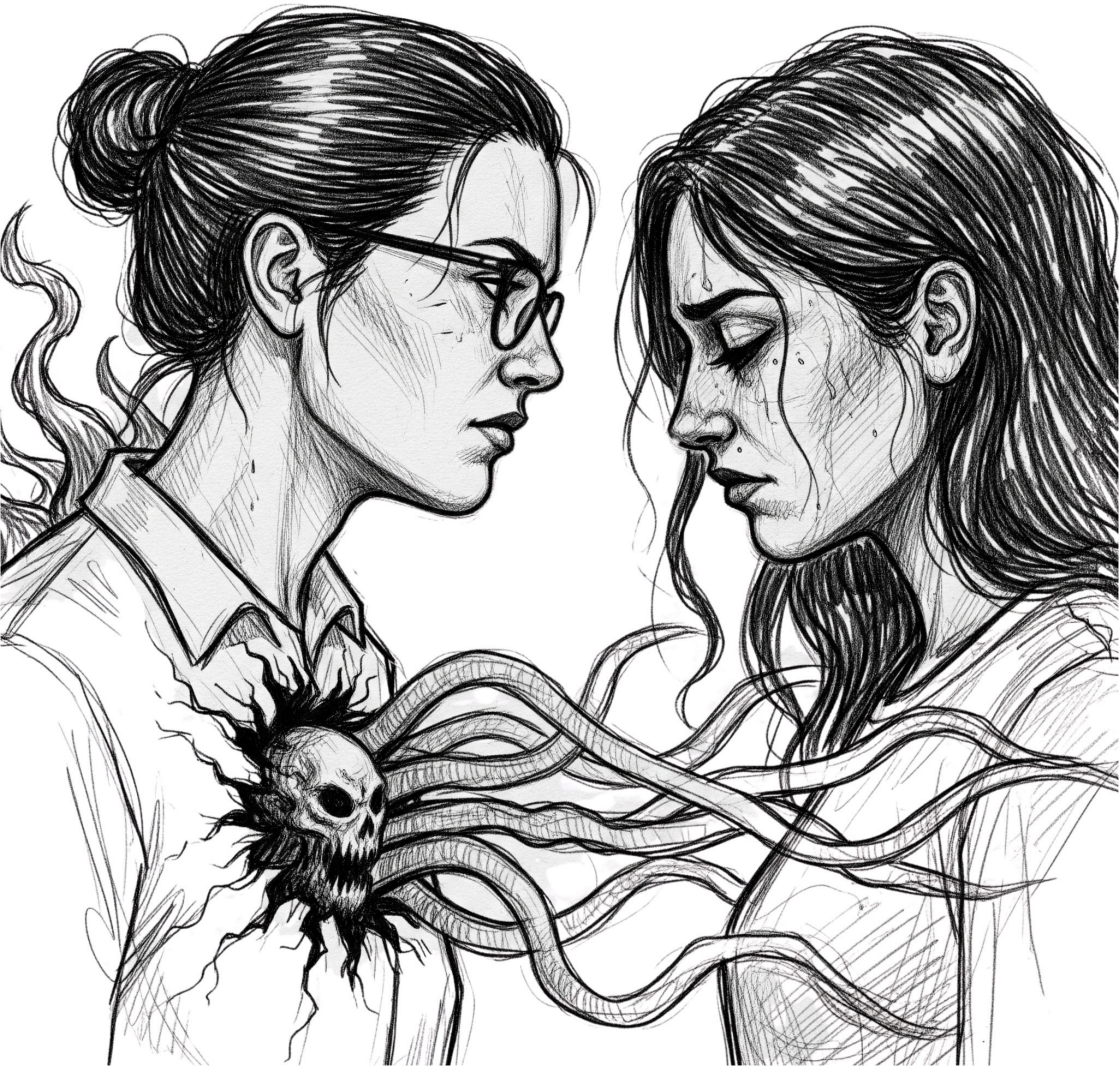
The aftermath of loving-hate: This figure visually represents the post-traumatic blurring of boundaries between intimacy and aggression. The illustration depicts the author with an Other and a *Monster* emerging from a black hole in the author’s chest. This aims to demonstrate the enduring legacy of enforced isolation, where the capacity to differentiate between love and harm has been damaged, leading to destructive relational patterns.

## Data Availability

The original contributions presented in the study are included in the article/supplementary material. Further inquiries can be directed to the corresponding author.

## Footnotes

1 While both terms refer to enforced isolation from meaningful human contact, they are distinct. In England, according to the Mental Health Act Code of Practice (2015) (5) seclusion refers to supervised confinement where a person is prevented from leaving - often a short-term crisis response. Long-term segregation (LTS) when applied to autistic people or people with learning disabilities refers to isolation lasting more than 48 hours (3) - generally a response to a perceived sustained risk of harm to others which is a constant feature of the persons presentation.

2 The terms 'Self' and 'Other' are capitalized to denote their specific meaning within psychoanalytic and relational theory. 'Self' refers to the core psychological structure and integrated sense of one's own subjectivity and identity, distinguishing it from the common pronoun. 'Other' refers to a distinct, separate subject with their own consciousness, from whom recognition is sought, rather than simply another person.
